# Supplementary material for: The Evolution and Characterization of the RNA Interference Pathways in Lophotrochozoa
Source: Genome Biol Evol. 2024 May 7;16(5):evae098. doi: 10.1093/gbe/evae098 (PMC11114477; doi:10.1093/gbe/evae098)
Supplement: evae098_Supplementary_Data [file evae098_supplementary_data.zip › Supplementary_materials.zip!/selected_miRNAs/NoGe_selected_miRNAs/mirdeep_pdf_files/NMRB01000449.1_Notospermus_geniculatus_strain_Ushimado_scaffold449,_whole_genome_shotgun_sequence_13613.pdf]

| | | |
|---|---|---|
| Provisional ID | : | NMRB01000449.1_Notospermus_geniculatus_strain_Ushimado_scaffold449,_whole_genome_shotgun_sequence_13613 |
| Score total | : | 29200.6 |
| Score for star read(s) | : | 3.9 |
| Score for read counts | : | 29192.2 |
| Score for mfe | : | 1.5 |
| Score for randfold | : | 0 |
| Score for cons. seed | : | 3 |
| Total read count | : | 57271 |
| Mature read count | : | 57255 |
| Loop read count | : | 0 |
| Star read count | : | 16 |

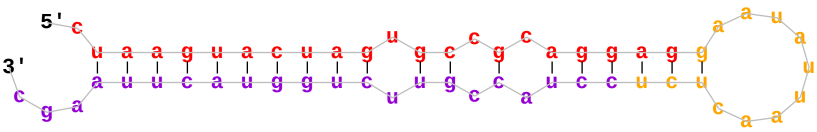


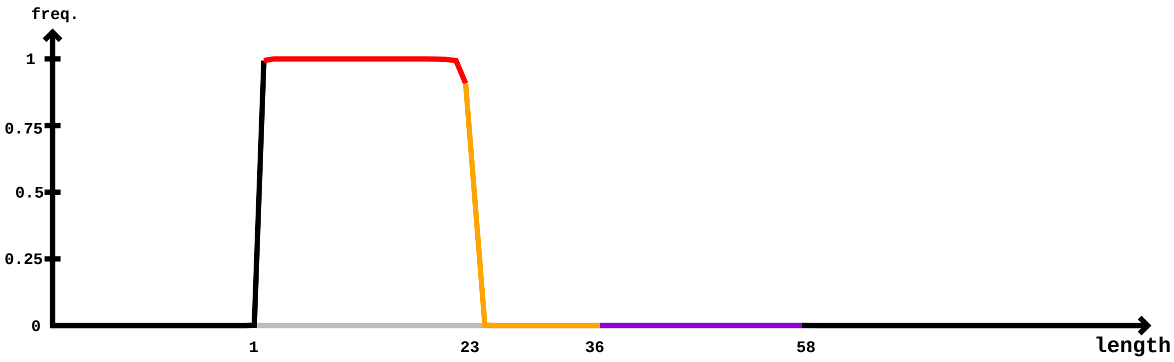


```
                         Mature                                     Star
5'- augagagccaucgucagauacuaaguacuagugccgcaggaggaauauuaacucuccuaccguucugguacuuaagcuaggacauggcgaacacaucucuggagggagcuga  -3'  obs
    augagagccaucgucagauacuaaguacuagugccgcaggaggaauauuaacucuccuaccguucugguacuuaagcuaggacauggcgaacacaucucuggagggagcuga       exp
    ......(((((.(((......((((((((((.((.(.((((((.........)))))).).)).))))))))))......)))))))).....((.((((....)))).)).   reads  mm  sample
    ...................acuaaguacuagugccgcagga.......................................................................   10     0   Fm3
    ...................acuaaguacuagugccgcaggaU......................................................................   1      1   Fm3
    ....................cuaaguacuagugccgca..........................................................................   11     0   Fm3
    ....................cuaaguacuagugccgUa..........................................................................   2      1   Fm3
    ....................cuaaguacuagugccgcaA.........................................................................   3      1   Fm3
    ....................cuaaguacuagugccgcaU.........................................................................   7      1   Fm3
    ....................cuaaguacuagugccgcag.........................................................................   21     0   Fm3
    ....................cuaaguacuagugccgcagA........................................................................   1      1   Fm3
    ....................cuaaguacuagugccgAagg........................................................................   1      1   Fm3
    ....................cuaaguacuagugccgcagg........................................................................   50     0   Fm3
    ....................cuaaguacuagugccgcagU........................................................................   5      1   Fm3
    ....................cuaaguacuaAugccgcagga.......................................................................   2      1   Fm3
    ....................cuaaguacuagugccgcGgga.......................................................................   4      1   Fm3
    ....................cuaagNacuagugccgcagga.......................................................................   1      1   Fm3
    ....................cuaUguacuagugccgcagga.......................................................................   1      1   Fm3
    ....................cuaaguacuagugccgcCgga.......................................................................   3      1   Fm3
    ....................cuaaguacuagugccgcagga.......................................................................   1207   0   Fm3
    ....................cuaaguacuagugcUgcagga.......................................................................   1      1   Fm3
    ....................cuaaguacuagugccgcUgga.......................................................................   4      1   Fm3
    ....................cuaagCacuagugccgcagga.......................................................................   1      1   Fm3
    ....................cuaaguacuagugccgAagga.......................................................................   11     1   Fm3
    ....................cCaaguacuagugccgcagga.......................................................................   1      1   Fm3
    ....................cuaaguacuagugccgcagUa.......................................................................   1      1   Fm3
    ....................cuaaguacuagugccgUagga.......................................................................   1      1   Fm3
    ....................cAaaguacuagugccgcagga.......................................................................   1      1   Fm3
    ....................Auaaguacuagugccgcagga.......................................................................   1      1   Fm3
    ....................cuaaguacuagugUcgcagga.......................................................................   4      1   Fm3
    ....................cuaaguacuGgugccgcagga.......................................................................   2      1   Fm3
    ....................cuaaguacuaUugccgcagga.......................................................................   2      1   Fm3
    ....................cuaaguacuaCugccgcagga.......................................................................   1      1   Fm3
    ....................cuaaguacuagugccgGagga.......................................................................   2      1   Fm3
    ....................cuaaguacuagugccgcaggU......................................................................   10     1   Fm3
    ....................cuUaguacuagugccgcaggag......................................................................   1      1   Fm3
```

Mature Star

```
augagagccaucgucagauacuaaguacuagugccgcaggaggaauauuaacucuccuaccguucugguacuuaagcuaggacauggcgaacacaucucuggagggagcuga
....................cuaaguacuagugccgcagAag......................................................................	1	1	Fm3
....................cuaaguUcuagugccgcaggag......................................................................	1	1	Fm3
....................cuaaguacuagugccgcaggag......................................................................	7600	0	Fm3
....................cuaaguacuagugccgcUggag......................................................................	26	1	Fm3
....................cAaaguacuagugccgcaggag......................................................................	4	1	Fm3
....................cuaaguaUuagugccgcaggag......................................................................	1	1	Fm3
....................cuaaguacuagugAcgcaggag......................................................................	2	1	Fm3
....................cuaaguacuagugccgcGggag......................................................................	23	1	Fm3
....................cuaaguacuagugccgUaggag......................................................................	18	1	Fm3
....................cuaaguacuagugccgcaggaC......................................................................	117	1	Fm3
....................Auaaguacuagugccgcaggag......................................................................	5	1	Fm3
....................cuaaguacuagugccgcagUag......................................................................	2	1	Fm3
....................cuaaguacuGgugccgcaggag......................................................................	1	1	Fm3
....................cuaaguacuaCugccgcaggag......................................................................	6	1	Fm3
....................Uuaaguacuagugccgcaggag......................................................................	5	1	Fm3
....................cuaaguacuagugccgGaggag......................................................................	6	1	Fm3
....................cuaaguacuagugccgAaggag......................................................................	27	1	Fm3
....................cuaaguacuagugccCcaggag......................................................................	1	1	Fm3
....................cGaaguacuagugccgcaggag......................................................................	2	1	Fm3
....................cuaaguaGuagugccgcaggag......................................................................	1	1	Fm3
....................cuaaguacuagugccgcCggag......................................................................	27	1	Fm3
....................cuaagNacuagugccgcaggag......................................................................	4	1	Fm3
....................cuaagCacuagugccgcaggag......................................................................	3	1	Fm3
....................cuaCguacuagugccgcaggag......................................................................	1	1	Fm3
....................cuaaguacuagugcGgcaggag......................................................................	1	1	Fm3
....................cuaagGacuagugccgcaggag......................................................................	1	1	Fm3
....................cuCaguacuagugccgcaggag......................................................................	2	1	Fm3
....................cuaaguacuagugccUcaggag......................................................................	1	1	Fm3
....................cuaaguacuagugUcgcaggag......................................................................	2	1	Fm3
....................cuaaguacuagugccgcaggaA......................................................................	23	1	Fm3
....................cCaaguacuagugccgcaggag......................................................................	3	1	Fm3
....................cuGaguacuagugccgcaggag......................................................................	2	1	Fm3
....................cuaaguacuaUugccgcaggag......................................................................	14	1	Fm3
....................cuaaguacuagugccgcaggUg......................................................................	1	1	Fm3
....................cuaagAacuagugccgcaggag......................................................................	1	1	Fm3
....................cuaaguacuaAugccgcaggag......................................................................	9	1	Fm3
....................cuaaguGcuagugccgcaggag......................................................................	1	1	Fm3
....................cuaaguacuagugccgcaCgag......................................................................	2	1	Fm3
....................cuaUguacuagugccgcaggag......................................................................	1	1	Fm3
....................Nuaaguacuagugccgcaggag......................................................................	6	1	Fm3
....................cuaaguacuagugcUgcaggag......................................................................	5	1	Fm3
....................cuaaguacuagugccgcaNgag......................................................................	1	1	Fm3
....................cuaaguacuagugccgcaggaU......................................................................	67	1	Fm3
....................cuGaguacuagugccgcaggagg.....................................................................	1	1	Fm3
....................cuaaguacuagugccgcCggagg.....................................................................	1	1	Fm3
....................cuaaguacuagugccgcaggagC.....................................................................	1	1	Fm3
....................cuaaguacuagugccgUaggagg.....................................................................	1	1	Fm3
....................cuaaguacuagugccgcaggagg.....................................................................	143	0	Fm3
....................cuaaguacuagugccgAaggagg.....................................................................	1	1	Fm3
....................cuaaguacuagugccgcaggagU.....................................................................	2028	1	Fm3
....................cuaaguacuagugccgcaggagA.....................................................................	363	1	Fm3
....................cuaaguacuaAugccgcaggagg.....................................................................	1	1	Fm3
....................cuaaguacuagugccgcaggaggU....................................................................	9	1	Fm3
....................cuaaguacuaguCccgcaggagga....................................................................	1	1	Fm3
....................cuaaguacuagugccgcaggagUa....................................................................	3	1	Fm3
....................cuaaguacuagugccgcaggagAa....................................................................	13	1	Fm3
.....................uaaguacuagugccgcag.........................................................................	3	0	Fm3
.....................uaaguacuagugccgcagg........................................................................	1	0	Fm3
.....................uaaguacuagugccgcagga.......................................................................	8	0	Fm3
.....................uaaguacuagugcGgcagga.......................................................................	1	1	Fm3
.....................uaaguacuagugccgcaggaC......................................................................	1	1	Fm3
.....................uaaguacuagugccgcCggag......................................................................	1	1	Fm3
.....................uaaguacuagugccgcaggaU......................................................................	3	1	Fm3
.....................uaaguacuagugccgcaggag......................................................................	37	0	Fm3
.....................uaaguacuagugccgcaggCg......................................................................	1	1	Fm3
.....................uaaguacuagugccgcaAgagg.....................................................................	1	1	Fm3
.....................uaaguacuagugccgcaggagg.....................................................................	7	0	Fm3
.....................uGaguacuagugccgcaggagg.....................................................................	3	1	Fm3
.....................uaaguacuagugccgcaggagU.....................................................................	13	1	Fm3
.....................uaaguacuagugccgcaggagga....................................................................	5	0	Fm3
```

**Mature** **Star**

| | | | |
|---|---|---|---|
| augagagccaucgucagauacuaaguacuagugccgcaggaggaauauuaacucuccuaccguucugguacuuaagcuaggacauggcgaacacaucucuggagggagcuga | | | |
| ........................guacuagugccgcaggag...................................................................... | 2 | 0 | Fm3 |
| .......................................................ccuaccguucugguacuuaagc................................... | 5 | 0 | Fm3 |
| .......................................................ccuaccguuAugguacuuaagc................................... | 1 | 1 | Fm3 |
| ........................................................cuaccguucugguacuuaagcu................................. | 3 | 0 | Fm3 |
| ...................acuaaguacuagugccgcagg........................................................................ | 2 | 0 | Mm3 |
| ...................acuaaguacuagugccgcagga....................................................................... | 2 | 0 | Mm3 |
| ...................Ccuaaguacuagugccgcaggag...................................................................... | 1 | 1 | Mm3 |
| ...................acuaaguacuagugccgcaggag...................................................................... | 1 | 0 | Mm3 |
| ...................acuaaguacuagugccgcaggaU...................................................................... | 1 | 1 | Mm3 |
| ...................acuaaguacuagugccgcaggagU..................................................................... | 1 | 1 | Mm3 |
| ....................cuaaguacuagugccgca.......................................................................... | 1 | 0 | Mm3 |
| ....................cuaaguacuagugccgcag......................................................................... | 8 | 0 | Mm3 |
| ....................cuaaguacuagugccgcagU........................................................................ | 1 | 1 | Mm3 |
| ....................cuaaguacuagugccgUagg........................................................................ | 1 | 1 | Mm3 |
| ....................cuaaguacuagugccgcagg........................................................................ | 51 | 0 | Mm3 |
| ....................cuaaAuacuagugccgcagg........................................................................ | 1 | 1 | Mm3 |
| ....................cuaaguacuagugccgcaggC....................................................................... | 2 | 1 | Mm3 |
| ....................Nuaaguacuagugccgcagga....................................................................... | 1 | 1 | Mm3 |
| ....................cuaaguacuagugccgcagga....................................................................... | 590 | 0 | Mm3 |
| ....................cuaaguacuaUugccgcagga....................................................................... | 1 | 1 | Mm3 |
| ....................cuaaguacuagugccgcUgga....................................................................... | 2 | 1 | Mm3 |
| ....................cuaaguacuagugccgcaggG....................................................................... | 3 | 1 | Mm3 |
| ....................cuaaguacuagugccgcCgga....................................................................... | 2 | 1 | Mm3 |
| ....................cuaaguacuaAugccgcagga....................................................................... | 1 | 1 | Mm3 |
| ....................cuaaAuacuagugccgcagga....................................................................... | 1 | 1 | Mm3 |
| ....................cuaaguacuagugccgGagga....................................................................... | 1 | 1 | Mm3 |
| ....................Uuaaguacuagugccgcagga....................................................................... | 1 | 1 | Mm3 |
| ....................cuaaguacuagugccgcaggU....................................................................... | 13 | 1 | Mm3 |
| ....................cuaaguacuaguAccgcagga....................................................................... | 1 | 1 | Mm3 |
| ....................cuaaguacuagugccgcagAag...................................................................... | 3 | 1 | Mm3 |
| ....................cuaaguacuagugccgcaggaU...................................................................... | 61 | 1 | Mm3 |
| ....................cuaaguacuagugccgUaggag...................................................................... | 14 | 1 | Mm3 |
| ....................cuaaguacuagugUcgcaggag...................................................................... | 2 | 1 | Mm3 |
| ....................cuaaguacuagugccgGaggag...................................................................... | 5 | 1 | Mm3 |
| ....................cuaaguacuagugccgcaAgag...................................................................... | 1 | 1 | Mm3 |
| ....................cuaaguacuagugccgcCggag...................................................................... | 16 | 1 | Mm3 |
| ....................cuaaguacuagugccgcaggag...................................................................... | 6598 | 0 | Mm3 |
| ....................cuCaguacuagugccgcaggag...................................................................... | 1 | 1 | Mm3 |
| ....................cuaaguacuaUugccgcaggag...................................................................... | 7 | 1 | Mm3 |
| ....................cuaaguacuaCugccgcaggag...................................................................... | 4 | 1 | Mm3 |
| ....................cuaaguacuagAgccgcaggag...................................................................... | 1 | 1 | Mm3 |
| ....................Uuaaguacuagugccgcaggag...................................................................... | 13 | 1 | Mm3 |
| ....................cuaaguUcuagugccgcaggag...................................................................... | 1 | 1 | Mm3 |
| ....................cuaaguacuagugccgcaggaA...................................................................... | 10 | 1 | Mm3 |
| ....................cuaaguacuagugccAcaggag...................................................................... | 1 | 1 | Mm3 |
| ....................cuaaguacuagugGcgcaggag...................................................................... | 1 | 1 | Mm3 |
| ....................cuaaguaAuagugccgcaggag...................................................................... | 1 | 1 | Mm3 |
| ....................cuaaguacuagugccgcaggGg...................................................................... | 4 | 1 | Mm3 |
| ....................cuUaguacuagugccgcaggag...................................................................... | 1 | 1 | Mm3 |
| ....................cuaaguacuUgugccgcaggag...................................................................... | 2 | 1 | Mm3 |
| ....................cuaagCacuagugccgcaggag...................................................................... | 1 | 1 | Mm3 |
| ....................cuaaguacuagugccgcaggCg...................................................................... | 3 | 1 | Mm3 |
| ....................cuaaguaUuagugccgcaggag...................................................................... | 2 | 1 | Mm3 |
| ....................cuaaguacuagugccgcGggag...................................................................... | 16 | 1 | Mm3 |
| ....................cuaaguacuagugccgAaggag...................................................................... | 29 | 1 | Mm3 |
| ....................cuaaguacAagugccgcaggag...................................................................... | 1 | 1 | Mm3 |
| ....................cuaaguacuagugccgcaggUg...................................................................... | 1 | 1 | Mm3 |
| ....................cuaaguacuaAugccgcaggag...................................................................... | 5 | 1 | Mm3 |
| ....................cuGaguacuagugccgcaggag...................................................................... | 6 | 1 | Mm3 |
| ....................cuaaguacuagugccgcUggag...................................................................... | 20 | 1 | Mm3 |
| ....................cuaaguacuagugccgcaggaC...................................................................... | 69 | 1 | Mm3 |
| ....................cuaaguGcuagugccgcaggag...................................................................... | 1 | 1 | Mm3 |
| ....................cuaCguacuagugccgcaggag...................................................................... | 1 | 1 | Mm3 |
| ....................cuaagAacuagugccgcaggag...................................................................... | 2 | 1 | Mm3 |
| ....................cuaaguacuaguCccgcaggag...................................................................... | 1 | 1 | Mm3 |
| ....................cAaaguacuagugccgcaggag...................................................................... | 5 | 1 | Mm3 |
| ....................Nuaaguacuagugccgcaggag...................................................................... | 6 | 1 | Mm3 |
| ....................cuaGguacuagugccgcaggag...................................................................... | 3 | 1 | Mm3 |
| ....................Nuaaguacuagugccgcaggagg..................................................................... | 1 | 1 | Mm3 |

Mature Star

| | | | |
|---|---|---|---|
| augagagccaucgucagauacuaaguacuagugccgcaggaggaauauuaacucuccuaccguucugguacuuaagcuaggacauggcgaacacaucucuggagggagcuga | | | |
| ....................cuaaguacuagugccgcaggagg..................................................................... | 76 | 0 | Mm3 |
| ....................cuaaguacuagugccgcCggagg..................................................................... | 1 | 1 | Mm3 |
| ....................cuaaguacuagugccgcaggagA..................................................................... | 180 | 1 | Mm3 |
| ....................cuaaguacuagugccgcaggagU..................................................................... | 4034 | 1 | Mm3 |
| ....................cuaaguacuagugccgcaggagC..................................................................... | 2 | 1 | Mm3 |
| ....................cuaaguacuagugccgcaggaggU.................................................................... | 16 | 1 | Mm3 |
| ....................cuaaguacuagugccgcaggagAa.................................................................... | 3 | 1 | Mm3 |
| .....................uaaguacuagugccgcagg........................................................................ | 2 | 0 | Mm3 |
| .....................uaaguacuagugccgcagga....................................................................... | 8 | 0 | Mm3 |
| .....................uaaguacuagugccgcaAga....................................................................... | 1 | 1 | Mm3 |
| .....................uaaguacuagugccgcaggaC...................................................................... | 1 | 1 | Mm3 |
| .....................uaaguacuagugccgcaggaU...................................................................... | 1 | 1 | Mm3 |
| .....................uaaguacuagugccgcaAgag...................................................................... | 1 | 1 | Mm3 |
| .....................uaaguacuagAgccgcaggag...................................................................... | 1 | 1 | Mm3 |
| .....................uaaguacuagugccgcaggag...................................................................... | 37 | 0 | Mm3 |
| .....................uaaguacuagugccgcaCgag...................................................................... | 1 | 1 | Mm3 |
| .....................uaaguacuagugccgcaUgag...................................................................... | 1 | 1 | Mm3 |
| .....................uaaguacuagugccgcaggagg..................................................................... | 4 | 0 | Mm3 |
| .....................uaaguacuagugccgcaggagU..................................................................... | 20 | 1 | Mm3 |
| .....................uaaguacuagugccgcaggagC..................................................................... | 1 | 1 | Mm3 |
| .....................uaaguacuagugccgcaggaggU.................................................................... | 1 | 1 | Mm3 |
| .......................................................ccuaccguucugguacuuaag.................................... | 1 | 0 | Mm3 |
| ....................cuaaguacuagugccgca.......................................................................... | 6 | 0 | Fm2 |
| ....................cuaaguacuagugccgcaA......................................................................... | 5 | 1 | Fm2 |
| ....................cuaaguacuCgugccgcagg........................................................................ | 5 | 1 | Fm2 |
| ....................cuaaguacuagugccgcagg........................................................................ | 32 | 0 | Fm2 |
| ....................cuaaguacuagugccgcCgga....................................................................... | 1 | 1 | Fm2 |
| ....................cuaaguacuaCugccgcagga....................................................................... | 1 | 1 | Fm2 |
| ....................cuaaguacuagugccgcUgga....................................................................... | 1 | 1 | Fm2 |
| ....................cuaaguacuagugccgcGgga....................................................................... | 1 | 1 | Fm2 |
| ....................cuaaguacuUgugccgcagga....................................................................... | 1 | 1 | Fm2 |
| ....................Auaaguacuagugccgcagga....................................................................... | 1 | 1 | Fm2 |
| ....................cuaaguacuagugccgcaggG....................................................................... | 2 | 1 | Fm2 |
| ....................cuaaguacuagugccgcagga....................................................................... | 292 | 0 | Fm2 |
| ....................Nuaaguacuagugccgcagga....................................................................... | 1 | 1 | Fm2 |
| ....................cuaaguacuagugccgcagUa....................................................................... | 1 | 1 | Fm2 |
| ....................cuaaguacuaUugccgcagga....................................................................... | 3 | 1 | Fm2 |
| ....................cuaaguacuaCugccgcaggag...................................................................... | 4 | 1 | Fm2 |
| ....................cuaaguacuagugccgcaggag...................................................................... | 3042 | 0 | Fm2 |
| ....................cuaaguacuagugccgcGggag...................................................................... | 8 | 1 | Fm2 |
| ....................cCaaguacuagugccgcaggag...................................................................... | 1 | 1 | Fm2 |
| ....................cuaaguacAagugccgcaggag...................................................................... | 1 | 1 | Fm2 |
| ....................cuaaguNcuagugccgcaggag...................................................................... | 2 | 1 | Fm2 |
| ....................cuaaguacuagugccgcagUag...................................................................... | 1 | 1 | Fm2 |
| ....................Uuaaguacuagugccgcaggag...................................................................... | 4 | 1 | Fm2 |
| ....................cuaaguacuagugccgGaggag...................................................................... | 2 | 1 | Fm2 |
| ....................cuaaguacuagugccgcagAag...................................................................... | 1 | 1 | Fm2 |
| ....................cuaaguacuagugccgcaggaU...................................................................... | 19 | 1 | Fm2 |
| ....................cuaaguacuagugccgcUggag...................................................................... | 11 | 1 | Fm2 |
| ....................cuaCguacuagugccgcaggag...................................................................... | 1 | 1 | Fm2 |
| ....................cAaaguacuagugccgcaggag...................................................................... | 1 | 1 | Fm2 |
| ....................cuaaguacuagugccgcaggaC...................................................................... | 44 | 1 | Fm2 |
| ....................cuaaguacuagugccgcaUgag...................................................................... | 1 | 1 | Fm2 |
| ....................cuaaguacuagCgccgcaggag...................................................................... | 1 | 1 | Fm2 |
| ....................cuaaguacuagugcUgcaggag...................................................................... | 2 | 1 | Fm2 |
| ....................Auaaguacuagugccgcaggag...................................................................... | 2 | 1 | Fm2 |
| ....................cuaaguacuagAgccgcaggag...................................................................... | 1 | 1 | Fm2 |
| ....................cuaaguacuagugccgcCggag...................................................................... | 8 | 1 | Fm2 |
| ....................cuaaguacuaUugccgcaggag...................................................................... | 4 | 1 | Fm2 |
| ....................cuaaguacuagugccgcaggaA...................................................................... | 6 | 1 | Fm2 |
| ....................cuaaguacuaAugccgcaggag...................................................................... | 1 | 1 | Fm2 |
| ....................cuaaguacuagugccgAaggag...................................................................... | 12 | 1 | Fm2 |
| ....................cuaagCacuagugccgcaggag...................................................................... | 1 | 1 | Fm2 |
| ....................cNaaguacuagugccgcaggag...................................................................... | 1 | 1 | Fm2 |
| ....................cuaaguacuagugccgUaggag...................................................................... | 4 | 1 | Fm2 |
| ....................Nuaaguacuagugccgcaggag...................................................................... | 1 | 1 | Fm2 |
| ....................cuaaguacuagugccgcCggagg..................................................................... | 2 | 1 | Fm2 |
| ....................cuaaguacuagugccgcaggagU..................................................................... | 3900 | 1 | Fm2 |
| ....................cuaaguacuagugccgcaggagA..................................................................... | 42 | 1 | Fm2 |

```
                    Mature                            Star
augagagccaucgucagauacuaaguacuagugccgcaggaggaauauuaacucuccuaccguucugguacuuaagcuaggacauggcgaacacaucucuggagggagcuga
....................cuaaguacuagugccgcaggagC.....................................................................  4     1  Fm2
....................cuaaguacuagugccgcaggagg.....................................................................  92    0  Fm2
....................cuaaguacuagugccgcaggaggU....................................................................  19    1  Fm2
....................cuaaguacuagugccgcaggaggG....................................................................  1     1  Fm2
.....................uaaguacuagugccgcaggag......................................................................  4     0  Fm2
.....................uaaguacuagugccgcaggagU.....................................................................  10    1  Fm2

...................acuaaguacuagugccUcagga.......................................................................  1     1  Fm1
...................acuaaguacuagugccgcagga.......................................................................  25    0  Fm1
...................acuaaguacuagugccgcaggaU......................................................................  11    1  Fm1
....................cuaaguacuagugccgcU..........................................................................  2     1  Fm1
....................cuaaguacuagugccgca..........................................................................  6     0  Fm1
....................cuaaguacuagugccgcCg.........................................................................  1     1  Fm1
....................cuaaguacuagugccgcaU.........................................................................  4     1  Fm1
....................cuaaguacuagugccgcag.........................................................................  6     0  Fm1
....................cuaaguacuagugccgcagg........................................................................  24    0  Fm1
....................cuaaguacuagugccgcUgg........................................................................  1     1  Fm1
....................cuaaguacuagugccgcagU........................................................................  6     1  Fm1
....................cuaaguacuagugccgGagg........................................................................  1     1  Fm1
....................cuaaguGcuagugccgcagga.......................................................................  1     1  Fm1
....................cuaaguacuagugccAcagga.......................................................................  1     1  Fm1
....................cuaaguacuagugccgcUgga.......................................................................  5     1  Fm1
....................cuaaguacuaAugccgcagga.......................................................................  1     1  Fm1
....................cuaaguacuagugccgcaggU.......................................................................  11    1  Fm1
....................cuaaguacuagugccgUagga.......................................................................  2     1  Fm1
....................cuaaguacuaCugccgcagga.......................................................................  1     1  Fm1
....................cuaaguacuagugccgcCgga.......................................................................  1     1  Fm1
....................cuaaguacuagugccgcagga.......................................................................  1331  0  Fm1
....................cuaaguacuagugccgGagga.......................................................................  1     1  Fm1
....................Nuaaguacuagugccgcagga.......................................................................  1     1  Fm1
....................cuaaguacuagugccgAagga.......................................................................  4     1  Fm1
....................cuaaguacuagugccgcaggG.......................................................................  7     1  Fm1
....................cuaaguacuagugccgcGgga.......................................................................  7     1  Fm1
....................Uuaaguacuagugccgcagga.......................................................................  1     1  Fm1
....................cuGaguacuagugccgcagga.......................................................................  2     1  Fm1
....................cuaaguacuagugUcgcagga.......................................................................  2     1  Fm1
....................cAaaguacuagugccgcagga.......................................................................  3     1  Fm1
....................cuaaguacuagugccgUaggag......................................................................  3     1  Fm1
....................cuaaguacuagGgccgcaggag......................................................................  1     1  Fm1
....................cuaaguacuaUugccgcaggag......................................................................  4     1  Fm1
....................cuaaguacuaAugccgcaggag......................................................................  9     1  Fm1
....................cuaaguacuaguAccgcaggag......................................................................  1     1  Fm1
....................Nuaaguacuagugccgcaggag......................................................................  2     1  Fm1
....................Uuaaguacuagugccgcaggag......................................................................  5     1  Fm1
....................cuaaguUcuagugccgcaggag......................................................................  2     1  Fm1
....................cuaaguacuagAgccgcaggag......................................................................  1     1  Fm1
....................cuaaguacuaCugccgcaggag......................................................................  2     1  Fm1
....................cAaaguacuagugccgcaggag......................................................................  1     1  Fm1
....................cuaaguaUuagugccgcaggag......................................................................  2     1  Fm1
....................cuaaguacuagugccgcaggaC......................................................................  37    1  Fm1
....................cuaaguacuagugcUgcaggag......................................................................  2     1  Fm1
....................cuaaguacuagugAcgcaggag......................................................................  1     1  Fm1
....................cuaaguacuagugccgcUggag......................................................................  9     1  Fm1
....................cuaaguacuagugccgcaggaU......................................................................  87    1  Fm1
....................cuaaguacuagugccgcaUgag......................................................................  1     1  Fm1
....................cuaaguacuagCgccgcaggag......................................................................  2     1  Fm1
....................cuaaguacuagugccgcaggag......................................................................  3405  0  Fm1
....................cuaagGacuagugccgcaggag......................................................................  1     1  Fm1
....................cuaaguacuagugccgcCggag......................................................................  20    1  Fm1
....................cuaaguacuagugccgAaggag......................................................................  15    1  Fm1
....................cuaaguGcuagugccgcaggag......................................................................  1     1  Fm1
....................cuaaguacuagugccgcaggaA......................................................................  3     1  Fm1
....................cuGaguacuagugccgcaggag......................................................................  1     1  Fm1
....................cuaaguacuaguUccgcaggag......................................................................  1     1  Fm1
....................cuaaguNcuagugccgcaggag......................................................................  1     1  Fm1
....................cuaaguacuagugccgcagUag......................................................................  2     1  Fm1
....................cuaaguacuagugccgcGggag......................................................................  13    1  Fm1
....................cCaaguacuagugccgcaggag......................................................................  2     1  Fm1
....................cuaaguacuagugccgGaggag......................................................................  4     1  Fm1
....................cuaaguacuUgugccgcaggag......................................................................  1     1  Fm1
```

Mature Star

```
augagagccaucgucagauacuaaguacuagugccgcaggaggaauauuaacucuccuaccguucugguacuuaagcuaggacauggcgaacacaucucuggagggagcuga
....................cuaaguacuagugccgcaggUg......................................................................   1     1   Fm1
....................cuaaguacuagugccgcaggagU.....................................................................   718   1   Fm1
....................cuaaguacuagugccgcaggagC.....................................................................   11    1   Fm1
....................cuaaguacuagugccgcaggagg.....................................................................   135   0   Fm1
....................cuaaguacuagugccgcaggaCg.....................................................................   1     1   Fm1
....................cuaaguacuagugccgcCggagg.....................................................................   2     1   Fm1
....................cuaaguacuagugccgcaggagA.....................................................................   219   1   Fm1
....................cuaaguacuagugccgAaggagg.....................................................................   3     1   Fm1
....................cuaaguacuagugccgcaggaggU....................................................................   14    1   Fm1
.....................uaaguacuagugccgcagg........................................................................   2     0   Fm1
.....................uaaguacuagGgccgcagga.......................................................................   1     1   Fm1
.....................uaaguacuagugccgcaCga.......................................................................   1     1   Fm1
.....................uaaguacuagugccgcagga.......................................................................   14    0   Fm1
.....................uaaguacuagugccgcaggag......................................................................   16    0   Fm1
.....................uaaguacuagugccgcCggag......................................................................   1     1   Fm1
.....................uaaguacuagugccgcaggagU.....................................................................   2     1   Fm1
.....................uaaguacuagugccgcaggagg.....................................................................   3     0   Fm1
.......................................................ccuaccguucugguacuuaagc...................................   5     0   Fm1

...................acuaaguacuagugccgcagga.......................................................................   5     0   Mm2
....................cuaaguacuagugccgca..........................................................................   3     0   Mm2
....................cuaaguacuagugccgcaU.........................................................................   3     1   Mm2
....................cuaaguacuagugccgcagg........................................................................   50    0   Mm2
....................cuaaguacuagugccgcagU........................................................................   2     1   Mm2
....................Uuaaguacuagugccgcagg........................................................................   1     1   Mm2
....................cuaaguacuagugccgcagC........................................................................   1     1   Mm2
....................cAaaguacuagugccgcagga.......................................................................   1     1   Mm2
....................cuaaguacuagugccgcCgga.......................................................................   2     1   Mm2
....................cuaaguacuagugccgcaggU.......................................................................   13    1   Mm2
....................cCaaguacuagugccgcagga.......................................................................   1     1   Mm2
....................cuaaguacuagugccgUagga.......................................................................   2     1   Mm2
....................cuaaguacuagugccgcaggG.......................................................................   10    1   Mm2
....................cuaaguacuagugccgcUgga.......................................................................   4     1   Mm2
....................cuaaguacuaCugccgcagga.......................................................................   1     1   Mm2
....................cuaaguacuaguCccgcagga.......................................................................   1     1   Mm2
....................cuaaguUcuagugccgcagga.......................................................................   1     1   Mm2
....................cuaaguacuagugccgcGgga.......................................................................   4     1   Mm2
....................cuaaguacuagugccgGagga.......................................................................   2     1   Mm2
....................cuaaguacuagugccgcagga.......................................................................   887   0   Mm2
....................cuaaguacuagugccgAagga.......................................................................   3     1   Mm2
....................cuaaguacuagugAcgcaggag......................................................................   2     1   Mm2
....................cuaCguacuagugccgcaggag......................................................................   1     1   Mm2
....................cuaaguacuagugccgcaggaA......................................................................   3     1   Mm2
....................cuaaguacuagugccgcaUgag......................................................................   1     1   Mm2
....................cuaaguUcuagugccgcaggag......................................................................   4     1   Mm2
....................cuUaguacuagugccgcaggag......................................................................   1     1   Mm2
....................cuaaguacuaAugccgcaggag......................................................................   7     1   Mm2
....................cuaaguacuaCugccgcaggag......................................................................   8     1   Mm2
....................cuGaguacuagugccgcaggag......................................................................   2     1   Mm2
....................cuaaguacuagugccgcaggGg......................................................................   3     1   Mm2
....................cuaaguacuagugccgcaggaU......................................................................   80    1   Mm2
....................Uuaaguacuagugccgcaggag......................................................................   11    1   Mm2
....................cuaaguacuagugccgcCggag......................................................................   13    1   Mm2
....................cuaaguacuagugccgcaggUg......................................................................   2     1   Mm2
....................cuaaguacuagugccgGaggag......................................................................   7     1   Mm2
....................cuaaguacuaUugccgcaggag......................................................................   6     1   Mm2
....................cuaaguacuagugccgcaggag......................................................................   6372  0   Mm2
....................cuaaguacuagugcGgcaggag......................................................................   2     1   Mm2
....................cuaaguacuagugccgcaggCg......................................................................   1     1   Mm2
....................cuaaguacuagugccgcGggag......................................................................   38    1   Mm2
....................cuaaguacuagugccgAaggag......................................................................   26    1   Mm2
....................cuaUguacuagugccgcaggag......................................................................   2     1   Mm2
....................cuaaguacuagugccgcUggag......................................................................   20    1   Mm2
....................cAaaguacuagugccgcaggag......................................................................   1     1   Mm2
....................cuaagCacuagugccgcaggag......................................................................   2     1   Mm2
....................cuaagAacuagugccgcaggag......................................................................   1     1   Mm2
....................cuaaguacuUgugccgcaggag......................................................................   2     1   Mm2
....................Nuaaguacuagugccgcaggag......................................................................   2     1   Mm2
....................cGaaguacuagugccgcaggag......................................................................   1     1   Mm2
....................cuaaguacuagCgccgcaggag......................................................................   1     1   Mm2
```

```
                    Mature                          Star
augagagccaucgucagauacuaaguacuagugccgcaggaggaauauuaacucuccuaccguucugguacuuaagcuaggacauggcgaacacaucucuggagggagcuga
....................cuaaguacuagugccgcaggaC......................................................................   59     1   Mm2
....................cuaaguacuagugccgUaggag......................................................................   16     1   Mm2
....................cuaaguacuagugcUgcaggag......................................................................   1      1   Mm2
....................cuaaguacuaCugccgcaggagg.....................................................................   1      1   Mm2
....................cuaaguacuagugccgUaggagg.....................................................................   1      1   Mm2
....................cuaaguacuagugccgcaggagg.....................................................................   139    0   Mm2
....................cuaaguacuagugccgcaggaCg.....................................................................   1      1   Mm2
....................cuaaguacuagugccgcaggagU.....................................................................   1826   1   Mm2
....................cuaaguacuagugccgcaggagA.....................................................................   142    1   Mm2
....................cuaaguacuagugccgcUggagg.....................................................................   1      1   Mm2
....................cuaaguacuagugccgcaggaggU....................................................................   7      1   Mm2
....................cuaaguacuagugccgcaggagAa....................................................................   1      1   Mm2
.....................uaaguacuagugccgcagga.......................................................................   7      0   Mm2
.....................uaaguacuagugccgcaggag......................................................................   33     0   Mm2
.....................uaaguacuagugccgcaggaU......................................................................   1      1   Mm2
.....................uaaguacuagugccgcaggagg.....................................................................   20     0   Mm2
.....................uaaguacuagugccgcaggagU.....................................................................   21     1   Mm2
.......................aguacuagugccgcaggag......................................................................   1      0   Mm2
.......................................................ccuaccguucugguacuuaagc...................................   1      0   Mm2

...................acuaaguacuagugccgcagga.......................................................................   1      0   Mm1
....................cuaaguacuagugccgca..........................................................................   5      0   Mm1
....................cuaaguacuagugccgcag.........................................................................   9      0   Mm1
....................cuaaguacuagugccgcagU........................................................................   9      1   Mm1
....................cuaaguacuagugccgcagg........................................................................   1      0   Mm1
....................cuaaguUcuagugccgcagga.......................................................................   1      1   Mm1
....................cuaaguacuagugccgcaggU.......................................................................   14     1   Mm1
....................cuaaguacuagugccgcCgga.......................................................................   3      1   Mm1
....................cuaaguacuaUugccgcagga.......................................................................   1      1   Mm1
....................cuaaguacuagugccgcGgga.......................................................................   1      1   Mm1
....................cuaaguacuagugccgcagga.......................................................................   293    0   Mm1
....................cuaaguacuagugccgcUgga.......................................................................   1      1   Mm1
....................cuaaguacuagugccgAagga.......................................................................   2      1   Mm1
....................cuaaguacuagugccgGaggag......................................................................   2      1   Mm1
....................cuaaguUcuagugccgcaggag......................................................................   1      1   Mm1
....................cuaaguacuagugccgcGggag......................................................................   14     1   Mm1
....................cuaaguCcuagugccgcaggag......................................................................   1      1   Mm1
....................cuaaguacuagugccgAaggag......................................................................   13     1   Mm1
....................cuaaguacuaCugccgcaggag......................................................................   2      1   Mm1
....................cuaagAacuagugccgcaggag......................................................................   1      1   Mm1
....................cCaaguacuagugccgcaggag......................................................................   1      1   Mm1
....................cuaaguacuagugAcgcaggag......................................................................   1      1   Mm1
....................cuaaguacuaAugccgcaggag......................................................................   5      1   Mm1
....................cAaaguacuagugccgcaggag......................................................................   1      1   Mm1
....................cuaaguacuagugccgUaggag......................................................................   6      1   Mm1
....................cuaaguacuagugccgcaUgag......................................................................   1      1   Mm1
....................cuaaguacuagugccgcCggag......................................................................   8      1   Mm1
....................cuaaguacuagugccgcaggaA......................................................................   15     1   Mm1
....................cuCaguacuagugccgcaggag......................................................................   1      1   Mm1
....................Uuaaguacuagugccgcaggag......................................................................   5      1   Mm1
....................cuUaguacuagugccgcaggag......................................................................   1      1   Mm1
....................cuaaguacuagugccgcaggaU......................................................................   11     1   Mm1
....................cuaaguacuagugccgcUggag......................................................................   9      1   Mm1
....................Nuaaguacuagugccgcaggag......................................................................   3      1   Mm1
....................cuaaguGcuagugccgcaggag......................................................................   1      1   Mm1
....................cuaaguacuaUugccgcaggag......................................................................   1      1   Mm1
....................cuaCguacuagugccgcaggag......................................................................   2      1   Mm1
....................cuaaguacuagugccgcaggag......................................................................   2957   0   Mm1
....................cuaaguacuagugccgcaggaC......................................................................   9      1   Mm1
....................cuaaguacuagugccgcaggagg.....................................................................   33     0   Mm1
....................cuaaguacuagugccgcaggagC.....................................................................   2      1   Mm1
....................cuaaguacuagugccgcaggagU.....................................................................   5899   1   Mm1
....................cuaaguacuagugccgcaggagA.....................................................................   30     1   Mm1
....................cuaaguacuagugccgcaggaggU....................................................................   16     1   Mm1
.....................uaaguacuagugccgcaggag......................................................................   9      0   Mm1
.....................uaaguacuagugccgcaggagU.....................................................................   30     1   Mm1
```
